# Optimalization of recovery, regeneration and rehabilitation

**DOI:** 10.1186/1878-5085-5-S1-A152

**Published:** 2014-02-11

**Authors:** Jaroslav Novak, Václav Zeman, Martin Matoulek, Ondřej Topolčan, Śárka Svobodova, Jaroslav Racek, Judita Kinkorova

**Affiliations:** 1 Medical Faculty Pilsen and Faculty Hospital Pilsen, Charles University Prague, Czech Republic; 2 1st Medical Faculty Prague and General Faculty Hospital Prague, Charles University, Prague, Czech Republic; 3 Technology Centre Academy of Sciences, Czech Republic

## Scientific objectives

Physically active subjects have superior aerobic performance and effort tolerance compared to the less active subjects. There was observed association between high exercise capacity and low mortality. The protective effect of physical activity and exercise capacity preventing the progression of diseases, and thus on premature death, could be one of explanations for this association. Most of this protection is assumed to result from the beneficial effects of physical exercise on blood lipids, blood pressure, glucose metabolism, vascular function, autonomic tone, blood coagulation, and inflammation.

## Technological approaches and results interpretation

We have currently initiated the examination of metabolic parameters, hormones, inflammatory parameters, growth angiogenic parameters and bone metabolism parameters in 4 groups of individuals: active sportsmen, sportsmen involved in sports with extreme load (i.e. ultra-marathon performance - e.g. 100 km running in 24 hrs, intermediate and long triathlons, long-distance swimming, etc.), in patients with cancer diseases and patients with cardiovascular diseases, who were individually recommended appropriate form of physical activity as a part of complex therapy

## Outlook and expert recommendations

The aim of this study was to compare the differences of the monitored parameter between the different groups of patients that are significantly different from health condition and from the level of physical activity. The final goal is to find the optimal biomarkers potentially useful as an indicator of optimal recovery course and rehabilitation after physical activity load – single evaluation and long term monitoring.

